# An IOHMM-Based Framework to Investigate Drift in Effectiveness of IoT-Based Systems

**DOI:** 10.3390/s21020527

**Published:** 2021-01-13

**Authors:** Gérald Rocher, Stéphane Lavirotte, Jean-Yves Tigli, Guillaume Cotte, Franck Dechavanne

**Affiliations:** 1 CNRS, Laboratoire I3S, Université Côte d’Azur (UCA), UMR 7271, 06900 Sophia Antipolis, France; stephane.lavirotte@univ-cotedazur.fr (S.L.); jean-yves.tigli@univ-cotedazur.fr (J.-Y.T.); franck.dechavanne@univ-cotedazur.fr (F.D.); 2 Telecom Physique, Université de Strasbourg, 67400 Illkirch-Graffenstaden, France; guillaume.cotte@etu.unistra.fr

**Keywords:** actuation, internet of things, ambient intelligence, cyber–physical systems, effectiveness, drift, input-output hidden markov models

## Abstract

IoT-based systems, when interacting with the physical environment through actuators, are complex systems difficult to model. Formal verification techniques carried out at design-time being often ineffective in this context, these systems have to be quantitatively evaluated for effectiveness at run-time, i.e., for the extent to which they behave as expected. This evaluation is achieved by confronting a model of the effects they should legitimately produce in different contexts to those observed in the field. However, this quantitative evaluation is not informative on the drifts in effectiveness, it does not help designers investigate their possible causes, increasing the time needed to resolve them. To address this problem, and assuming that models of legitimate behavior can be described by means of Input-Output Hidden Markov Models (IOHMMs), a novel generic unsupervised clustering-based IOHMM structure and parameters learning algorithm is developed. This algorithm is first used to learn a model of legitimate behavior. Then, a model of the observed behavior is learned from observations gathered in the field. A second algorithm builds a dissimilarity graph that makes clear structural and parametric differences between both models, thus providing guidance to designers to help them investigate possible causes of drift in effectiveness. The approach is validated on a real world dataset collected in a smart home.

## 1. Introduction

Systems based on the Internet of Things (IoT-based systems) such as ambient assisted living systems (AAL) [1] and smart cyber–physical systems (CPS) [2], by interacting with the physical environment through actuators, face many challenges, pertaining to *reliability*, *safety* and *resilience* requirements. These challenges are difficult to meet because the physical environment is *complex* and difficult to model [3]. Designers are thus limited in their ability to predict the effects of their interactions at design-time and to formally verify their conformity to logical properties, whether functional or temporal. It is then necessary to quantitatively assess the *effectiveness* of these systems at run-time [4], i.e., the extent to which these systems behave as expected. This assessment is carried out by confronting a model of the effects legitimately expected to be produced in particular contexts to those observed in the field [5]. This model of legitimate behavior is generally built from the experience of experts, documents describing standards and safety, users’ preferences, etc., which are formulated by If-Then rules for their interpretation [6].

The quantitative effectiveness assessment, as *quality* (e.g., *quality of service* (QoS) [7,8], *quality of experience* (QoE) [9]), *performance* and *reliability* assessment metrics (e.g., time-to-failure (TTF), remaining useful life (RUL), etc.), can be used to highlight any deterioration of an IoT-based system and trigger analyses leading to corrective actions. However, these assessments do not provide guidance to designers that would help them direct their research and identify the possible causes of drifts in effectiveness, increasing the time needed to resolve them. More specifically, drifts in effectiveness, from the model of the legitimate behavior perspective, can be threefold: (1) the system does not behave as expected (2), the model is *incomplete*, the observed behavior, although legitimate, has not been foreseen and (3), there is an *incipient drift in parameters*, i.e., the observed behavior, although legitimate, is slightly outside the expectations (also known as concept drift). Whether it is due to unforeseen anomalous or legitimate behaviors or to parameters drifting over time, designers must be provided with tools to support them in investigating drifts in effectiveness by correlating various events and consulting system experts to possibly determine their corresponding root causes as well as their relevant data, thereby reducing the time needed to resolve them.

**Figure 1:**
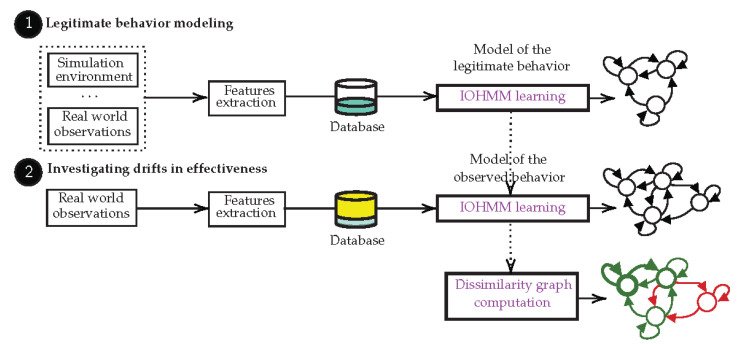
Steps to implement the proposed approach.

In this paper, we consider IoT-based systems whose model of the legitimate behavior can be described with Input-Output Hidden Markov Models (IOHMMs) [10]. This framework is widely used for behavioral modeling [11,12] and brings numerous advantages herein [13]; (1) it is an explainable graphical model [14] part of the Dynamic Bayesian Networks (DBN) family; (2) it formalizes conditional dependencies between the effects and their stimuli (i.e., *contextual input*, *events*); (3) it incorporates tolerances on expectations. On the basis of this assumption, the contribution of this paper is two-fold:

1. With the assumption that observations are stochastically correlated with the IoT-based system conditions (i.e., there exists a *bijection* between states and observations [15]), we develop a novel generic clustering-based algorithm for learning both the IOHMM structure Φ (states and state-transitions) and the parameters λ (distribution parameters) from continuous observations. The algorithm is proposed to be implemented with the *Hierarchical Density-Based Spatial Clustering of Applications with Noise* (HDBSCAN*) [16] clustering algorithm and a recent incremental extension denoted by *Flexible, Incremental, Scalable, Hierarchical Density-Based Clustering for Arbitrary Data and Distance* (FISHDBC) [17].
2. On the basis of this algorithm, we propose a framework for investigating drifts in effectiveness of IoT-based systems. This framework takes place in two steps as depicted in Figure 1:
  (a) The learning algorithm is fed with observations corresponding to the effects expected to be produced by an IoT-based system, leading to the learning of a model of the legitimate behavior. This model can further be used to quantitatively assess the effectiveness of the system at run-time as done in [5].
  (b) This model is incremented with observations of the effects produced by the system in operation within its environment, leading to the learning of a model of the observed behavior. Then, on the basis of these models, an algorithm is proposed and used to build a *directed dissimilarity graph* that highlights differences between both models, thereby helping designers direct their research and identify the possible causes of drifts in effectiveness.

## 2. Related Works

The problems addressed in this paper have connections with the fields of model-based *fault diagnosis* as well as *fault detection and identification* and *system health monitoring*, just to name a few.

In [18], authors apply IOHMM to failure diagnosis, prognosis and health monitoring of a diesel generator. The model is used to classify observations so as to determine the degradation state of the system. The model contains three states, a first one characterizing the legitimate behavior, a second one characterizing the presence of a degradation and a third one characterizing a severe degradation. This approach is commonly used in the literature [19,20] where Markovian models formalize legitimate and anomalous behaviors, often grouped into abstract states whose semantics result from an analysis carried out upfront. Faults identification and detection is then similar to a classification problem (i.e., estimation of the current state, be it legitimate or anomalous).

**One of the advantages of these approaches is that HMMs (and IOHMMs) are explainable models [14]. However, while these approaches are relevant when the state space of the considered systems is limited, they are not (at best hardly) applicable to complex systems whose set of anomalous (and legitimate) states cannot be completely known upfront [21]**.

In order to detect unknown emerging behaviors, whether legitimate or anomalous, some authors envisage models with variable state spaces on the basis of unsupervised online learning schemes. For instance, in [22], authors developed an incremental online model learning approach that extends the *BIRCH clustering algorithm* [23]. While the model grows continuously as new data arrive, it has also the capability to forget obsolete observations. In [24], authors investigated the *Typicality and Eccentricity Data Analytics (TEDA)* clustering approach [25], based on density estimates. In addition to these approaches, some authors use adaptive HMMs to assess the health condition of mechanical systems. In [26], a method in the Statistical Process Control (SPC) is combined with an adaptive HMM for unknown wear states detection and diagnosis of a turning process. The structure of the model is changed to represent degradation processes in the presence of unknown faults. In [27], an adaptive HMM is used for online learning of dynamic changes in the health of machines. New health states are described online by adding new hidden states in the HMM. Health degradations are quantified online by a Health Index (HI) that measures the similarity between two density distributions that respectively describe the historic and current health states.

**While the aforementioned approaches allow to increment models with unforeseen legitimate or anomalous behaviors, they do not provide guidance to designers to help them investigate possible causes of the anomalous behaviors**. For instance, in [22], anomalous behaviors trigger alarms, incremental learning being justified for reducing the false alarm rate. In [24], designers are provided with a quantitative evaluation, not informative on the symptoms and the causes of the anomalous behaviors.

Interesting approaches have been implemented as part of the QoE. For instance, in [28], the authors use the *Pseudo Subjective Quality Assessment* (PSQA) approach to automatically assess the QoE in the field of video streaming. The PSQA (also known as Quantitative Quality Assessment, QQA) approach consists of learning, by performing a set of subjective tests, how humans react to quality. The learning tool used is a specific class of neural network (Random Neural Network, RNN). The QoE assessment is then based on an estimation function Q defined during the learning phase and specific features in video streams (e.g., the frame loss rate or the effective bandwidth of the connection). In [29], a systematic online health assessment approach is proposed on the basis of a Growing Hierarchical Self-Organizing Map (GHSOM) algorithm, a variant of Self-Organizing Map (SOM), a type of Artificial Neural Network (ANN), with adaptive self-learning techniques. The method enables the identification of novel working condition states, such as new rotating speed or processing recipe, and the recognition of new degradation extent in the arriving monitoring data, and includes them into the prior learning models.

**RNNs can model a wide range of dynamical systems [30]. However, they have a large number of parameters making them hardly interpretable and explainable [31] and impractical for providing guidance to designers to help them investigate drifts.** In [29], although SOM produce low-dimensional, discretized representation of the observation space, authors do not explicitly describe in what form engineers and operators are kept informed of any significant change in the model.

Specific to IoT, in [32], a systematic literature review has been conducted on the developed statistical and machine learning methods for anomaly detection, analysis and prediction in smart environments, transportation networks, health care systems, smart objects and industrial systems. **In this study, a gap was found in the visualization of anomalies for the analysis. The authors recognize that new methods and approaches are needed in order to analyze anomalies.** The approach proposed in this document aims at filling this gap. It is based on the IOHMM modeling framework, which makes it possible to define explainable behavioral models. Like some of the aforementioned approaches, we envisage models with variable state spaces on the basis of unsupervised online learning schemes for learning the behavior of a system from observations. However, unlike these approaches, we propose an algorithm that, beyond metrics, makes clear the differences between the model of the legitimate behavior and the one learned from observations, providing guidance to designers to help them investigate anomalous behaviors.

## 3. Background on Input-Output Hidden Markov Model

**Figure 2:**
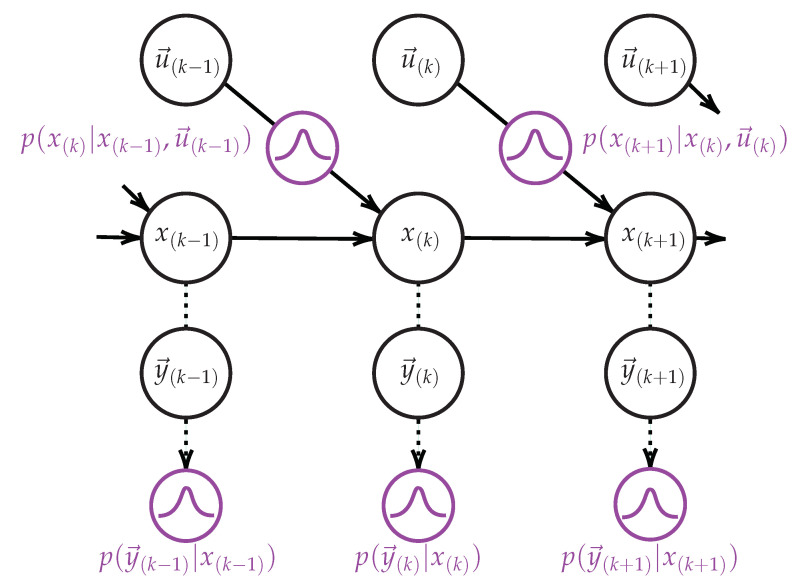
Bayesian network expressing conditional dependencies of an Input-Output Hidden Markov Model (IOHMM). The model is said ”hidden” because the states of the processes they model are not directly observable but inferred from contextual input $\vec{u}$ output $\vec{y}$.

In this paper, we consider IoT-based systems whose model of the legitimate behavior can be described with the IOHMM modeling framework [10]. IOHMMs model a pair of stochastic input-output processes (U,Y) as a result of an underlying stochastic Markovian process X that cannot be observed (it is said hidden). From a generative point of view, a sequence of input-output continuous observations ${\left({\vec{u}}_{\left(k\right)},{\vec{y}}_{\left(k\right)}\right)}_{k=1}^{K},K\in N^{🟉}$ ($N^{🟉}$ is the set of positive natural numbers greater than 0), ${\vec{u}}_{\left(k\right)}\in R^{n}$ ($R^{n}$ is an n-dimensional vector of real numbers), ${\vec{y}}_{\left(k\right)}\in R^{m}$, is the outcome of a path along the states of X; ${\vec{y}}_{\left(k\right)}$ and ${\vec{u}}_{\left(k\right)}$ are instances of the random variables $\left(U_{\left(k\right)},Y_{\left(k\right)}\right)$ whose distributions are respectively governed by density functions determined by the states and the state transitions along the path. Formally, a continuous density discrete state space (discrete time) Input-Output Hidden Markov Model (IOHMM), whose graphical representation is depicted in Figure 2, is defined by the tuple $<Q,\vec{\pi },A,\vec{B}>$ where:

- $Q=\left\{x_{1},x_{2},\ldots ,x_{N}\right\}$ is the finite set of hidden states; $x_{\left(k\right)}$ denotes the hidden state at time *k*,
- $\vec{\pi }={\left(\pi_{1},\pi_{2},\ldots ,\pi_{N}\right)}^{T}$ is the initial state distribution vector. $\pi_{i}$ denotes the likelihood of the state *i* to be the first state of a state sequence. In this paper, we assume that the elements $\pi_{i}$ of $\vec{\pi }$ are equally probable, i.e., equal to $\frac{1}{N}$,
- *A* is the N×N state transition matrix, where each element $a_{ij}$ of the matrix is a *n*-dimensional contextual input distribution (1≤i,j≤N). Thus, $a_{ij}\left(\vec{u}\right)=p\left(x_{\left(k+1\right)}=j|x_{\left(k\right)}=i,{\vec{u}}_{\left(k\right)}=\vec{u}\right)$ denotes the likelihood of transitioning to state $x_{\left(k+1\right)}=j$ at time k+1, given the current state $x_{\left(k\right)}=i$ and the contextual input vector ${\vec{u}}_{\left(k\right)}=\vec{u}\in R^{n}$ at time *k*,
- $\vec{B}={\left(b_{1},b_{2},\ldots ,b_{N}\right)}^{T}$ is the state emission vector, where each element $b_{i}$ (1≤i≤N) is a *m*-dimensional output distribution. $b_{i}\left(\vec{y}\right)=p\left({\vec{y}}_{\left(k\right)}=\vec{y}|x_{\left(k\right)}=i\right)$ denotes the likelihood of observing the output vector ${\vec{y}}_{\left(k\right)}=\vec{y}\in R^{m}$ at time *k* while being in the state $x_{\left(k\right)}=i$. The output observation ${\vec{y}}_{\left(k\right)}$ at time *k* only depends on the state $x_{\left(k\right)}$ at time *k*.

The structure Φ of an IOHMM is defined by the number of states *N* and the elements $a_{ij}$ of the state transition matrix *A* such that there exists an input $\vec{u}$ that leads a transition from the state *i* to the state *j* (i.e., ∃$\vec{u}$ s.t. $a_{ij}\left(\vec{u}\right)>0$, ∀1≤i,j<N). The parameters λ of an IOHMM correspond to the input and output distribution parameters.

This model serves as a basis for efficient solutions to several inference problems [33]:

(1) The problem of inferring the likelihood of an observation sequence (i.e., $p\left({\left({\vec{u}}_{\left(k\right)},{\vec{y}}_{\left(k\right)}\right)}_{k=1}^{K}\right)$), as well as inferring the distribution over hidden states at the end of the observation sequence (i.e., $p\left(x_{\left(K\right)}|\left({\vec{u}}_{\left(1\right)},{\vec{y}}_{\left(1\right)}\right),\ldots ,\left({\vec{u}}_{\left(K\right)},{\vec{y}}_{\left(K\right)}\right)\right)$) can be solved by the Forward algorithm (filtering).
(2) The problem of inferring the distribution over hidden states anywhere in the observation sequence (i.e., $p\left(x_{\left(k\right)}|\left({\vec{u}}_{\left(1\right)},{\vec{y}}_{\left(1\right)}\right),\ldots ,\left({\vec{u}}_{\left(K\right)},{\vec{y}}_{\left(K\right)}\right)\right)$, k<K) can be solved by the Forward-Backward algorithm (smoothing).
(3) The problem of inferring the most likely sequence of hidden states that led to the generation of the observation sequence can be solved by the Viterbi algorithm.

This modeling framework brings numerous advantages [13]; (1) it is an explainable graphical model, there is a 1:1 correspondence between observations and states; (2) it formalizes conditional dependencies between the effects (outputs) and their context (inputs), making them suitable to model dynamical systems; (3) it incorporates tolerances on expectations related to uncertainties inherent in the natural variability of physical processes and disturbances possibly resulting from adaptation mechanisms [34] (randomness [35]) and/or uncertainties related to prior knowledge on the system and/or users’ expectations (epistemic uncertainties [5,36]).

## 4. IOHMM Structure and Parameters Learning

The approach proposed in this paper considers the modeling of the legitimate behavior of an IoT-based system as an IOHMM and compares this model with that learned from field observations. The objective was to highlight the differences in behavior that may appear during the operation of the system. To achieve this, the proposed approach thus requires an IOHMM model to be learned from observations prior considering comparing both models and highlighting their differences, i.e., it requires to learn the IOHMM structure and parameters from field observations. While structure and parameters learning problem has been intensively studied in the case of HMMs, **no solution has been proposed to date within the IOHMM framework apart from learning the parameters [11,37,38]. The algorithm proposed in the sequel is then a novel algorithm**. As part of the solutions proposed within the HMM framework, *local search* algorithms [39] start from an initial guess of the structure Φ and iterate, by adding, removing states and transitions and by reversing state transitions, until reaching the structure that maximizes the Bayes Information Criterion (BIC). *State merging* algorithms [40] (conceptually, very similar to *agglomerative clustering* algorithms) first build a maximum likelihood structure Φ where a different state is associated to each of the observations and where transitions relative to consecutive states (i.e., consecutive observations) are assigned a probability of 1 while the others are assigned a probability of 0. Then, at each iteration, the structure Φ is modified by merging the states on the basis of the maximization of the posterior Bayesian probability criterion. Other approaches have been studied in the literature. For instance, in [41], authors propose an algorithm using both state splitting and state merging operations. In [42], authors incrementally construct the structure Φ using an *Instantaneous Topological Map* (ITM) algorithm [43]. Here, the hidden state space is continuous and supposed to be *discretizable* into a finite number of observable regions, every region being represented by a discrete state in the HMM.

Structure learning, whether through local search or state merging, consists in empirically estimating the best state space segmentation from data. In all the aforementioned approaches, parameters λ are learned by using an *Incremental Expectation-Maximization* (EM) clustering algorithm (e.g., Baum-Welch [44]). Underlying this algorithm is the assumption that observations can be modeled by *Gaussian mixture models* (GMMs) [45] where parameters λ are described by the mean and the covariance matrix. In the case of multimodal likelihood functions, however, there is no guarantee that the algorithm will avoid becoming trapped at a local maximum, resulting in an inferior clustering solution [46].

In the sequel, we propose a novel unsupervised generic algorithm for learning IOHMM structure Φ and parameters λ. It is generic in the sense that it can accommodate any continuous space clustering algorithm and is not limited to GMMs.

### 4.1. A Generic Unsupervised Clustering-Based IOHMM Learning Algorithm

What characterizes discrete state space IOHMMs and derivatives is that they model stochastic processes whose states are hidden, i.e., they can only be inferred from continuous observation sequences. Learning the structure Φ and parameters λ (i.e., *identifying* the model) is first and foremost about segmenting the observation space into a finite number of relevant *regions* such that each region represents a discrete state, i.e., it is assumed that observations are stochastically correlated with the system conditions, thereby taking advantage of understanding its structure [47]. A region, in this context, refers to *"a state of nature that governs the observation generation process. It can be viewed as a source of observations whose distribution is governed by a density function specific to the region"* [48].

Observation space segmentation into regions can be achieved using unsupervised *clustering* algorithms. *“These algorithms try to group observations so that the regions thereby obtained reflect the different generation processes they are governed by”* [48]. On this basis, we consider a two steps generic algorithm for learning the structure Φ and parameters λ of first order IOHMMs from continuous observation sequences ${\left({\vec{u}}_{\left(k\right)},{\vec{y}}_{\left(k\right)}\right)}_{k=1}^{K}$. The proposed algorithm is described hereafter (Algorithm 1).
**Algorithm 1:** Learning of IOHMM structure Φ and parameters λ.
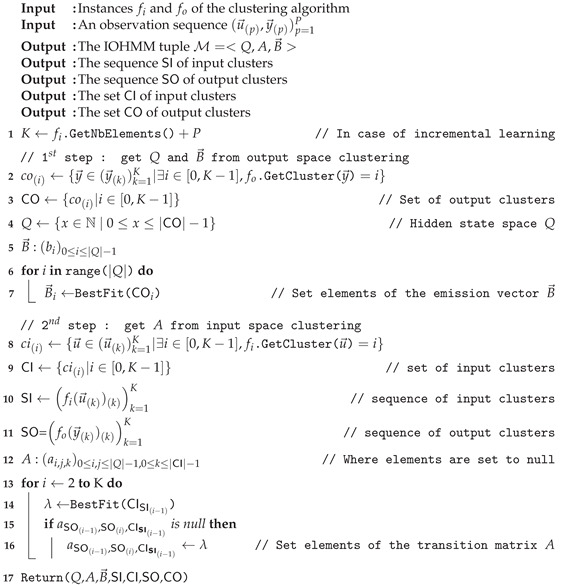


The first step consists of segmenting the output observation space ${\left({\vec{y}}_{\left(k\right)}\right)}_{k=1}^{K}$ into regions (corresponding discrete states in the model) using a clustering algorithm defined by the function $f_{o}:R^{m}\mapsto N^{🟉}$. Each region is associated with all output observations belonging to it ($co_{\left(i\right)}$, line 2). The set of output regions CO (line 3) provides us with *Q* (line 4) and the elements $b_{i}$ of the vector $\vec{B}$ (i.e., the output distribution parameters) are computed by fitting the set $co_{\left(i\right)}$ of output observations associated with the region *i* into the most appropriate (multivariate) distribution (line 7) (With the number of states given by *Q*, one can alternatively compute the parameters λ using an Expectation-Maximization algorithm, though it assumes observations whose distributions are Gaussian).

The second step consists of segmenting the input observation space ${\left({\vec{u}}_{\left(k\right)}\right)}_{k=1}^{K}$ into regions using a clustering algorithm defined by the function $f_{i}:R^{n}\mapsto N^{🟉}$. Here again, each input observation is associated with the region it belongs to ($ci_{\left(i\right)}$, line 8). The set of input regions is defined by CI (line 9). Then, input and output observation spaces are classified according to the identified regions, i.e., each observation in the sequences ${\left({\vec{u}}_{\left(k\right)}\right)}_{k=1}^{K}$ and ${\left({\vec{y}}_{\left(k\right)}\right)}_{k=1}^{K}$ is associated to its corresponding region. One obtains the sequence of input regions (SI line 10) and the sequence of states (SO line 11) from which is built the state transition matrix *A*. The sequence of states SO determines elements $a_{ij}$ to be populated (lines 15 and 16) with input distribution parameters (line 14). Here, each state transition is allowed to handle multiple distributions. Although not allowed in the standard (probabilistic) IOHMM model, this will be useful in investigating the differences between legitimate and observed behaviors (see Section 5).

Let us consider the example depicted in the table below:
**k****1****2****3****4****5****6****7****8****9*****…*****SI**111222200*…***SO***…*000111122

Output observations first belong to the state 0 (as defined for k=2 in SO), then there are state transitions from the state 0 to the state 0 (k:2→3 and k:3→4), then a state transition from the state 0 to the state 1 (k:4→5), then from the state 1 to the state 1 (k:5→6, k:6→7 and k:7→8), etc. Following this sequence of states, elements $a_{00}$, $a_{01}$, $a_{11}$, $a_{12}$ and $a_{22}$ have to be populated with input distribution parameters. Recall from the IOHMM model that the state at time *k* depends on the input at time k−1. Thus,$a_{k-1,k}≜{\mathrm{CI}}_{{\mathrm{SI}}_{\left(k-1\right)}}$, i.e., $a_{k-1,k}$ contains the distribution parameters obtained by fitting the set of input observations associated with the input region ${\mathrm{SI}}_{\left(k-1\right)}$ at time k−1 into most appropriate (multivariate) distribution. Thus, considering k:2→3, $a_{00}$ contains distribution parameters associated with input observations of the input region 1 (as defined in SI at time k=2), $a_{01}$ and $a_{11}$ contain those associated with input observations of the input region 2, etc.

As such, the proposed algorithm accommodates any unsupervised (possibly incremental) clustering algorithm where incremental has to be understood here as the ability to process continuous observation sequences as they arrive [49]. In this paper, we assume the following hypotheses:

1. The number of states |Q| is not known a priori.
2. The processes underlying the observations are unknown, so neither their distribution, their density, nor their law of generation (shape) are known.
3. Observations could be noisy and, considering incremental learning, incomplete.

So as to address these hypotheses, we consider the HDBSCAN* hierarchical density-based clustering algorithm [16].

### 4.2. Implementation with HDBSCAN*

The ${\mathrm{HDBSCAN}}^{🟉}$ algorithm [16], and its incremental extension (FISHDBC [17]), is a clustering algorithm for exploratory data analysis that extends the *Density-Based Spatial Clustering of Applications with Noise* (DBSCAN) algorithm [50]. It can operate correctly up to 100 dimensional data (https://hdbscan.readthedocs.io/en/latest/faq.html).

In the context of this paper, the main advantages of the algorithm lie in the fact that:

1. It does not require to specify upfront the number of clusters in the data.
2. It can find non-linearly separable clusters of varying shapes and densities.
3. The ordering of the data does not matter.
4. It supports *outlier* (or noise) assignments as being observations isolated in sparse regions.

**Figure 3:**
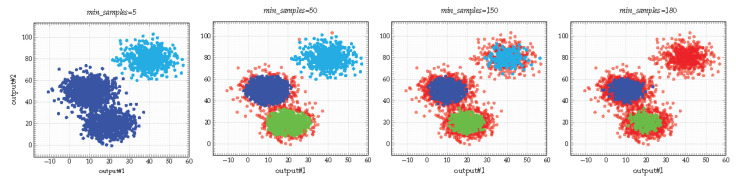
Example of clustering results for different values of *min_samples* (total obs = 2624). The larger the value of *min_samples*, the more conservative the clustering, i.e., clusters will be restricted to progressively more dense regions (outliers are depicted in red).

Among the aforementioned advantages, the notion of outliers is particularly relevant when considering model learning. Let us consider two types of outliers:

1. *Intrinsic outliers* depend on how *conservative* one wants to be in learning the model, governed by the HDBSCAN* *min_samples* parameter. The larger the value of *min_samples*, the more conservative the clustering, i.e., clusters will be restricted to progressively more dense regions with, as a consequence, a higher number of outliers (https://hdbscan.readthedocs.io/en/latest/parameter_selection.html) (Figure 3). The notion of conservatism can be related to that of *distinguishability*, i.e., the structure of an IOHMM is distinguishable when all the distributions (elements of the transition matrix A and the emission vector $\vec{B}$) are pairwise distinct, supporting a *bijection* between states and observations [15]. On the contrary, the structure of an IOHMM is completely hidden when all observation distributions are equal thereby, it is clearly impossible to distinguish between states and inference of the structure is impossible. In between these two extrema, increasing the *min_samples* parameter thus helps making observation distributions pairwise distinct (see Figure 3),
2. *Extrinsic outliers* depend on the observations (noise) and are particularly interesting in the context of incremental learning. Indeed, while observation sequences arrive progressively, some might reinforce the density of an existing region while some others might form a sparse region not yet dense enough to be considered as a cluster (i.e., there is a lack of knowledge leading these observations to be *temporarily* considered as outliers and discounted from the clustering process).

Whether intrinsic or extrinsic, outliers have to be discounted from the clustering process. Assuming that they are associated to a dummy cluster $c_{\left(i\right)}$, i<0, Algorithm 1 is modified as described in Algorithm 2.
**Algorithm 2:** Outliers removal (extension of Algorithm 1).
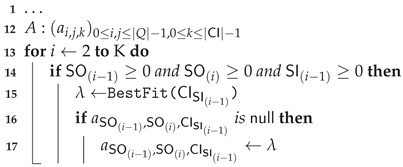


### 4.3. Experimental Evaluation

**Figure 4:**
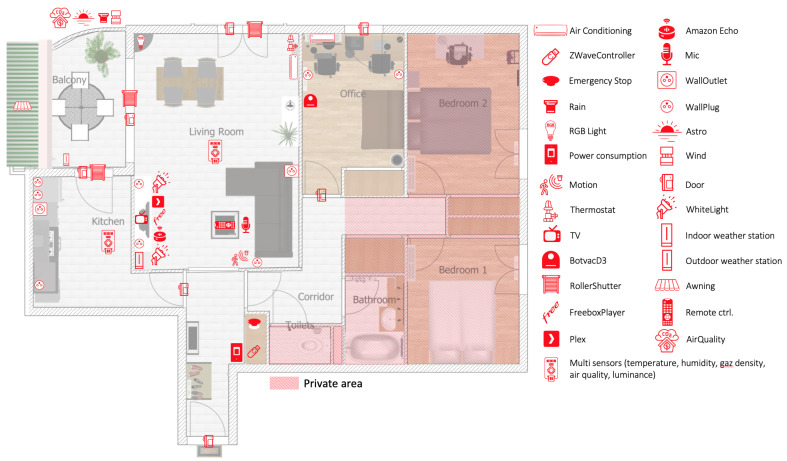
Experimentation setup.

**Figure 5:**
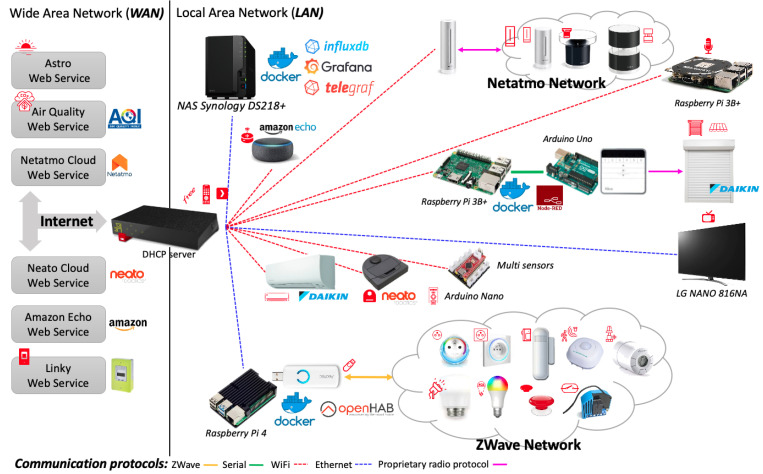
IoT infrastructure.

The experimentation is carried out on a real world dataset gathered in a smart-home equipped with 54 IoT devices offering up to 228 sensors and actuators (Figure 4). The associated infrastructure is depicted in the Figure 5. Several IoT protocols are used to communicate with all devices (ZWave, Wifi, etc.). Computational resources (Arduino Uno/Nano, Raspberry Pi) are made available at the edge of the infrastructure where software components are deployed through docker containers. These software components define the control logic for actuators (e.g., roller shutters) or extract features from sensor observations (e.g., the sound characteristics of the microphone signal). Observations are recorded into a database (InfluxDB) on a regular basis. For instance, ZWave devices provide one observation per minute, Netatmo devices provide observations each 10 min, the status of the Neato smart vacuum cleaner is updated each minute, etc.

On the basis of this setup, the objective is to learn a model of the legitimate behavior of a set of devices in different contexts on the basis of sound features. The devices considered for this experiment are the TV and the Amazon Echo smart speaker.

From a modeling perspective, output observations (legitimate effects) are characterized by Mel-Frequency Cepstral Coeffs (MFCCs) and Zero Crossing Rate (ZCR) sound features, extracted from a microphone signal and processed at the edge of the IoT infrastructure by a Raspberry Pi. Input observations (contexts) are characterized by the operating status of the TV and the Amazon Echo smart speaker. MFCC and ZCR features have been selected for their capacity at producing a good segmentation of the observation space.

**Figure 6:**
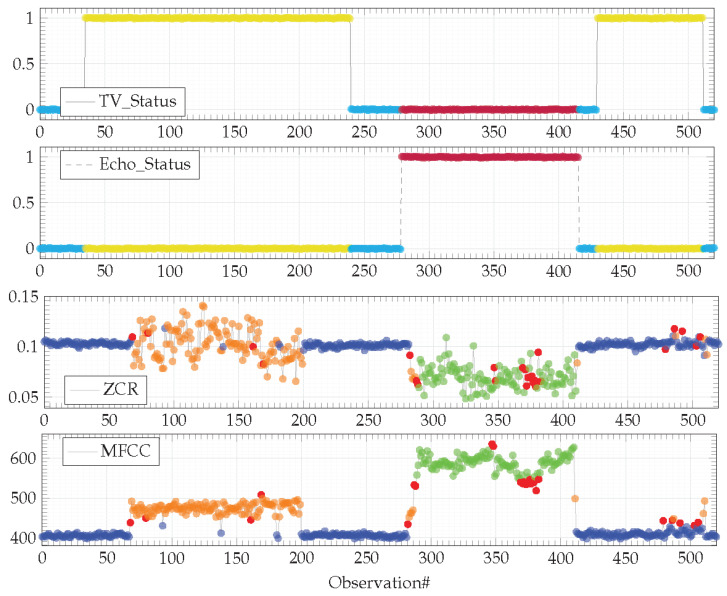
Real dataset representing the sound effects legitimately produced by two devices (TV and Amazon Echo smart speaker) in different contexts. Observations are colored according to the clusters identified by the HDBSCAN* algorithm with *min_samples* = 40.

While both devices can produce sound into the environment, users expect that they do not produce it simultaneously. The dataset used for the experiment reproduces this legitimate behavior as depicted in Figure 6.

Observations are colored according to the contexts identified by the HDBSCAN* algorithm with *min_samples* = 40. Input observations (i.e., TV_Status and Echo_Status) are segmented into three clusters. Observations are colored in cyan when both devices are switched OFF. Observations are colored in yellow when the TV is ON and the Amazon Echo smart speaker is OFF. Finally, observations are colored in magenta when the TV is OFF and the Amazon Echo smart speaker is OFF. Output observations (i.e., ZCR and MFCC sound features) are also segmented into three clusters. The blue one corresponds to the silence; the orange one characterizes the sound emitted by the TV and the green one characterizes the sound emitted by the Amazon Echo smart player (here, playing music). As expected, none of the devices operate simultaneously.

**Figure 7:**
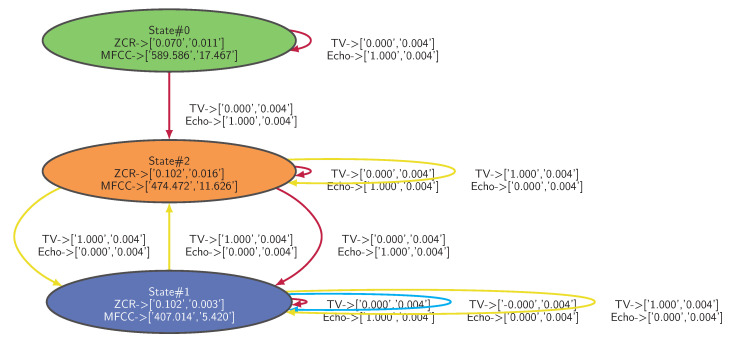
IOHMM model of the legitimate behavior learned from the input/output observations depicted in Figure 6 applied to Algorithm 1 (*min_samples* = 40).

The dataset is applied to the Algorithm 1 implemented with HDBSCAN* with *min_samples* parameter set to 40. The IOHMM model of the legitimate behavior learned from the dataset is depicted in Figure 7 where distributions are fitted into Gaussian Mixture Models (GMMs), characterized by the mean and the standard deviation of each feature (i.e., <feature> -> [<mean>,<stdev>]).

The completeness of the IOHMM model learned here depends, however, on the input-output observations the algorithm has been fed with during the learning process. There is no certainty that the model is complete, a legitimate behavior may occur in the long run that was not part of the initial set of observations. For instance, what if a new device producing sound is added into the environment? A new state should appear, whether legitimate or not, characterizing the sound emitted by this new device. Generally speaking, the behavior observed in the field in the long run may no longer correspond to that which is supposed to be legitimate, defined at a certain point in time. Whether this is due to the fact that the model of the legitimate behavior is incomplete (e.g., not enough observations), that its parameter values do not fit the concrete legitimate behavior anymore (e.g., due to the drift in the system parameters) or that new legitimate or illegitimate states and/or state transitions have appeared, it is necessary to provide designers with tools that allow them to investigate and understand changes that have potentially occurred.

## 5. Investigating Drifts in Effectiveness

In this paper, the study of drifts in effectiveness is carried out by identifying the *structural and parametric dissimilarities* between the model of the legitimate behavior of an IoT-based system and a model learned from field observations. In the literature, dissimilarities between two HMMs are mainly characterized by a *measure*. In [51], authors present a method that computes a variant of the Hellinger distance between two HMMs representing legitimate and observed behaviors. In [52], authors use the Wasserstein distance. While these quantitative assessments might be useful as performance indicators, they do not explain, however, the structural and parametric differences between the models. Moreover, the aforementioned measures provide a global distance between the distributions of probability thereby, are limited to only provide dissimilarities on the parameters.

In the sequel, we propose an algorithm that builds a dissimilarity graph between legitimate and observed behaviors, making clear both structural and parametric differences and providing guidance to designers to help them investigate possible causes of drift in effectiveness, reducing the time needed to resolve them.

### 5.1. An Algorithm for Identifying IOHMMs Structural and Parametric Dissimilarities

The approach proposed for identifying IOHMMs structural and parametric dissimilarities is based on the learning Algorithm 1, implemented with the FISHDBC algorithm [17], a recent incremental extension of the HDBSCAN* clustering algorithm. The main idea (Algorithm A2) is first to learn, using the learning Algorithm 1 implemented with FISHDBC, a model of the legitimate behavior of the IoT-based system from input-output observations, either generated from simulations or gathered from the field. Then, at some point in time, field observations, gathered on the long run, are incrementally fed into the algorithm. The following scenarios, denoting dissimilarities between legitimate and observed behaviors, may occur:

1. (**Structure related**) Output observations gathered from the field lead new clusters to occur. These new clusters define new states (either legitimate or anomalous) not anticipated/foreseen in the model of the legitimate behavior.
2. (**Structure related**) Input observations gathered from the field lead new state transitions to occur (either legitimate or anomalous) not anticipated/foreseen in the model of the legitimate behavior.
3. (**Parameters related**) Input and output observations gathered from the field lead distribution parameters (e.g., mean and standard deviation) to be slightly modified, denoting a drift in the initial parameter values.
4. (**Structure and Parameters related**) Input-output observations gathered from the field do not cover the states and state transitions defined in the model of the legitimate behavior. This situation implies that either not enough observations have been gathered from the field (e.g., rare events) or that the model is somehow wrong, i.e., it expects a different behavior than the one concretely implemented.

IOHMMs being graphical models one can highlight the aforementioned dissimilarities, thereby helping designers to identify changes that may have occurred. To this end, the algorithm described in Appendix A builds a dissimilarity graph as follows:

1. (lines 3 and 4) First, using the Algorithm 1 implemented with FISHDBC clustering algorithm, a model of the legitimate behavior is learned from observations ${\left({\vec{u}}_{\left(v\right)},{\vec{y}}_{\left(v\right)}\right)}_{v=1}^{V}$. Then, this model is incremented with observations ${\left({\vec{u}}_{\left(w\right)},{\vec{y}}_{\left(w\right)}\right)}_{w=1}^{W}$ gathered from the field in the long run.
2. (line 5) On the basis of the sets of clusters CI and CO and the sequences of clusters SI and SO obtained from the previous step, one can compute, using the Algorithm A1, the vector ${\vec{B}}_{w}$ and the matrix $A_{w}$ of the frequency of occurrence of each state and state transition, respectively.
3. (lines 6 to 16) The first *V* elements of SO correspond to the expected states computed from ${\left({\vec{y}}_{\left(v\right)}\right)}_{v=1}^{V}$. The remaining V+W+1 elements correspond to the states obtained from output observations ${\left({\vec{y}}_{\left(w\right)}\right)}_{w=1}^{W}$ gathered from the field. The idea is to parse states associated to the latter and verify if they are present or not in the former. A node is created in the dissimilarity graph for each state whose color depends on whether the state is present (blue, meaning the state is expected) or not (red, the state is not expected). The reverse process is done (lines 13 to 16) to detect states that are present in the former but not present in the latter (orange, an expected state is not covered). The width of the states depends on their frequency of occurrence given by ${\vec{B}}_{w}$.
4. (lines 17 to 21) This part of the algorithm is devoted to compute the transition matrix $A^{\prime }$ corresponding to the first *V* elements of SI and SO corresponding to the legitimate behavior.
5. (lines 22 to 30) Transition matrices *A* and $A^{\prime }$ are compared for dissimilarities. Edges are added into the dissimilarity graph for each state transition whose color depends on whether a legitimate (blue) or illegitimate (red) state transition occurred. Legitimate state transitions not covered are associated to edges colored in orange in the dissimilarity graph. The width of the state transitions depends on their frequency of occurrence given by $A_{w}$.

### 5.2. Experimental Evaluation

**Figure 8:**
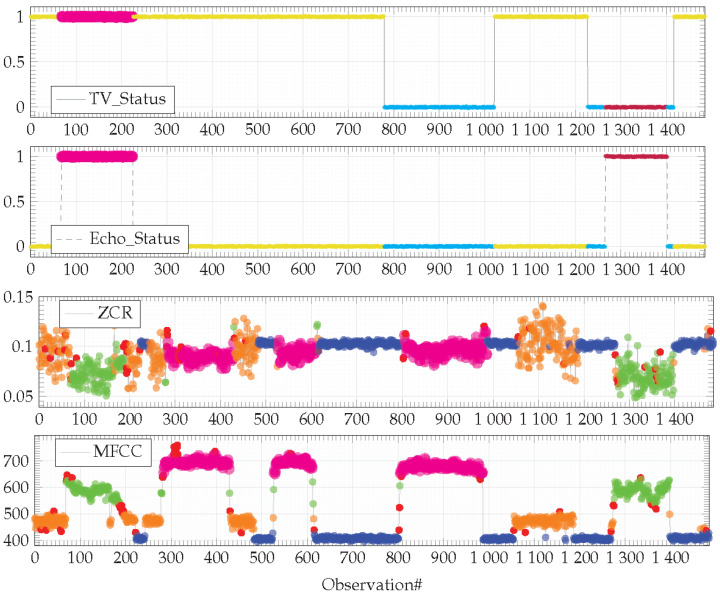
Observations from the field colored according to the clusters identified by the FISHDBC algorithm with *min_samples* = 40. Observations colored in magenta denote behaviors not anticipated/foreseen in the model of the legitimate behavior depicted in Figure 7, learned on the basis of the dataset depicted in Figure 6.

The dataset used in the Section 4.3, corresponding to the legitimate behavior of an IoT-based system is complemented with longer run observations gathered from the same physical environment (Figure 8).

This dataset highlights two unforeseen behaviors (denoted in magenta in Figure 8) that will have to be highlighted by the dissimilarity graph algorithm described in the previous section. A first behavior concerns a configuration on the TV and the Amazon Echo smart speaker, both operating simultaneously (Figure 8, input features from observation#80 to #230). This behavior is illegitimate, both devices must not operate simultaneously. The second behavior is related to a new device that has appeared in the environment. This device is an autonomous smart vacuum cleaner the inhabitants have recently acquired (Figure 8, output features from observation#280 to #430, then from observation#520 to #610 and, finally, from observation#800 to #1000). This behavior is legitimate, the model of the legitimate behavior has to be updated according to this new device.

**Figure 9:**
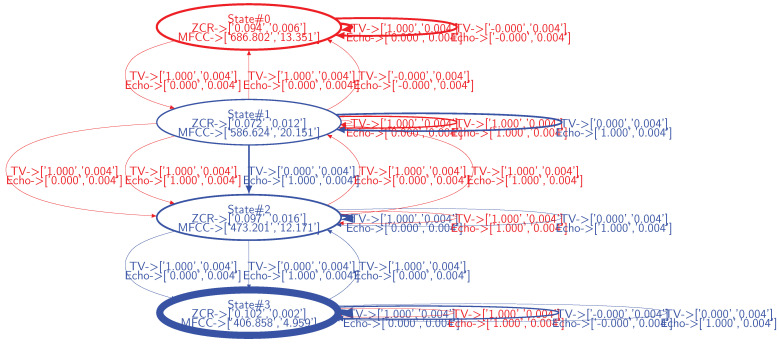
Dissimilarity graph between the legitimate behavior whose model is depicted in Figure 7 and the behavior observed from field observations, depicted in Figure 8. States and state transitions colored in red denote differences between both models (*min_samples* = 40).

The dataset representing the legitimate behavior (Figure 6) and the dataset representing the behavior observed from the field (Figure 8) are applied to the Algorithm A2. The resulting dissimilarity graph is depicted in Figure 9.

Observations from the field whose behavior corresponds to the legitimate behavior lead blue states and state transitions to appear, while those whose behavior is not legitimate or legitimate but unforeseen in the model, lead red states and state transitions to appear. For instance, the state 0 in Figure 9 corresponds to the smart vacuum cleaner emitting noise while being in operation. This state is not part of the model of the legitimate behavior, i.e., ZCR/MFCC observations characterizing this state do not correspond to any state defined in the model of the legitimate behavior. Thus, according to the model of the legitimate behavior, this device is not supposed to exist. Thus, a drift in effectiveness occurs as soon as the smart vacuum cleaner enters the environment. In this case, the model of the legitimate behavior must be updated with this legitimate state as well as with the operating status of the vacuum cleaner as a condition for entering/exiting this new state. As such, the dissimilarity graph provides the symptoms of drifts in effectiveness, not their root cause. From the designers’ point of view, investigations must be carried out, leading to correlate, in this case, the ZCR/MFCC observation values with the smart vacuum cleaner.

In addition to this new state, additional state transitions occur, denoting unforeseen legitimate and illegitimate behaviors. For instance, state transitions occur where both the TV and the Amazon Echo smart speaker operate simultaneously (denoted in magenta in Figure 8, TV_Status and Echo_Status are set to one). This behavior is not legitimate (users expect that these devices do not produce sound simultaneously) and, from a designer perspective, the root cause analysis is straightforward. It should be noted that such a configuration does not lead to the emergence of a new state. Actually, from the selected sound features, the configuration where both devices operate simultaneously leads to oscillate between state 1, state 2 and state 3 characterizing the TV in operation, the Amazon Echo smart speaker in operation and the silence, respectively. In this context, the IOHMM modeling framework leveraged in this paper, by allowing to specify contextual inputs, adds valuable information that help designers to analyze drifts in effectiveness.

Additional state transitions occur where either the TV or the Amazon Echo smart speaker are in operation. These configurations are legitimate but were not part of the data used to learn the model of the legitimate behavior.

The proposed algorithm enables statistical analysis on states and state transitions, i.e., the width of the states and the state transitions depends on their frequency of occurrence. For instance, the state 3 (corresponding to the silence) in Figure 9, occurs more frequently than others. Providing designers with this information may add valuable insights on the behavior of the system beyond dissimilarities between states and state transitions.

## 6. Conclusions and Perspectives

IoT-based systems whose purpose is achieved through interactions with the physical environment are complex and difficult to model. State-of-the-art formal verification techniques carried out at design-time are often ineffective even though the trustworthiness of these systems remains a first class concern. One way to address this problem is to quantitatively evaluate their effectiveness at run-time on the basis of a model of their legitimate behavior and field observations. However, while this quantitative evaluation can be leveraged as part of a monitoring process, thereby triggering actions in the field and/or conceptual investigations on the system in response to drifts in effectiveness, it does not provide guidance to designers to help them investigate their possible causes, increasing the time needed to resolve them. To address this problem, a novel unsupervised generic clustering-based Input-Output Hidden Markov Model (IOHMM) structure and parameters learning algorithm, implemented with HDBSCAN*/FISHDBC clustering algorithms was presented. These algorithms were first leveraged to learn the model of the legitimate behavior of an IoT-based system from continuous observations either generated from simulations or gathered from the field. This model was then complemented with observations from the field, characterizing the IoT-based system in operation. Then, a second algorithm was proposed to generate a dissimilarity graph making it clear to designers the structural and parametric differences between both models, helping them to investigate their possible causes. In [32], a gap was found in the visualization of anomalies for the analysis of IoT-based systems. The authors recognize that new methods and approaches are needed. The approach proposed in this paper contributes to this field.

The approach proposed in this paper is generic; assuming that observations are stochastically correlated with the IoT-based system conditions, (1) the unsupervised learning algorithm can accommodate any continuous observation space clustering algorithm, (2) observations can be fitted into any distribution type beyond Gaussian Mixture Models (GMM) usually assumed, for instance, in the Expectation-Maximization-based (EM) algorithms (e.g., Baum-Welch).

Although promising, the approach presented in this paper raises questions that shall be addressed in future research. For instance, specific to the HDBSCAN* algorithm and derivatives is the question on how to choose the *min_samples* parameter. A possible approach would be to handle model learning as an optimization problem where the *min_samples* parameter value chosen is the one maximizing a particular criteria (e.g., likelihood, Bayesian Information Criteria (BIC)). The IOHMM unsupervised learning algorithm presented in this paper is based on observation space clustering and we also plan to leverage clustering evaluation indexes (e.g., *silhouette* [53], *Davies-Bouldin* [54] or *Calinski-Harabasz* [55]) as criteria. Finally, we plan to implement a functionality that would enable the learning algorithm to forget the obsolete elements of the IOHMM model learned from field observations.

## Data Availability

Publicly available datasets were analyzed in this study. This data can be found here: https://gitlab.com/enact/behavioural_drift_analysis/-/blob/master/demos/demo_smarthome/Dataset_MDPI_Sensors.zip.

## Appendix A. Dissimilarity Graph Computation Algorithms

**Algorithm A1:** States and state transitions weights computation.

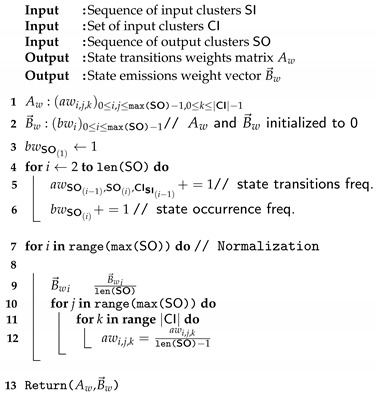

**Algorithm A2:** Dissimilarity graph computation.
